# Ten simple rules for more objective decision-making

**DOI:** 10.1371/journal.pcbi.1007706

**Published:** 2020-04-02

**Authors:** Anthony C. Fletcher, Georges A. Wagner, Philip E. Bourne

**Affiliations:** 1 Retired, London, United Kingdom; 2 Department of Mechanical Engineering, University of Melbourne, Victoria, Australia; 3 School of Data Science, University of Virginia, Charlottesville, Virginia, United States of America; Dassault Systemes BIOVIA, UNITED STATES

## Introduction

Scientists spend their lives analyzing data by the systematic study of the structure and behavior of the physical and natural world using both observation and experiment—objective analysis. But when it comes to decision-making, scientists are also humans with accompanying subjectivity. Put colloquially, we have both heart and head—and are capable of being simultaneously subjective and objective.

Here we posit that bringing more objectivity ("head") to decisions is a good thing. It's a key part of "critical thinking," the "Socratic questioning" method. We are not suggesting, that like Mr. Spock, we should be driven entirely by rationality, nor are we considering the merits of various reasoning systems [1]; we are simply examining why greater objectivity helps in providing a simple way to achieve improved objectivity. So, to start, is objectivity indeed better than subjectivity?

To address this question, it’s useful to look at the 2 opposite ends of the spectrum: objectivity is really the application of pure logic (something is either right or wrong, more or less, etc.), whereas subjectivity [2] is embodied in the form of what is often called Cartesian Doubt or skepticism (that the knowledge of anything outside ones direct experience has to be considered as unsure). In certain cases, increased objectivity is superior, for example, when the decision being taken leads toward a measurable or quantifiable outcome: if there is a specific goal in mind, then it's very useful to be able to estimate how close that decision might get you to that goal before you set out on the path. In real life, most decisions are a mixture of head and heart, but with these rules, we hope to increase both the accuracy and quantity of the head part while not neglecting the heart.

But enough of the epistemological concepts, what we want is to make better decisions (better here being more objective) and look at 10 ways in which we might do this, culminating in a simple tool that anyone with a spreadsheet (or even a pen and paper) can use.

Each rule is accompanied by a use case, some drawn from 2 previous Ten simple rules: Ten simple rules for graduate students [3] and Ten simple rules for selecting a postdoctoral position [4].

We will culminate with a worked example that illustrates this approach. Every lab needs a good coffee machine, and we are inspired by the example of the famous Trojan Room coffee pot. Based in the old computer laboratory of the University of Cambridge, England, in 1991, it provided the inspiration for the world's first webcam [5]. So here we show how to make sure your coffee is well up to par!

## Rule 1: Break decisions into smaller parts

Most decisions involve a range of issues that all need to be weighed up, not only in a like-for-like comparison, but also considering nonsimilar elements. The idiom about comparing apples to oranges serves this well: yes, they are clearly different, but they also have lots of similarities (you can compare the calorific content, the "mouth feel", nutrient make-up, etc.), Ultimately, you might like the taste of one but not the other (which is essentially subjective) or you might need to stave off scurvy (which is objective), but all things being equal, you could compare the characteristics and see whether one is preferable to the other under a given situation.

Thus, we can break down the apple versus orange decision into smaller parts (elements) as fully objective comparisons, subjective comparisons, and some that might be termed quasi-objective, in that they can rely on third party or expert opinion being nonpartisan and at least derived, in part, from a desire to be data-driven. Example studies comparing apples and oranges have been published by both NASA [6] and in the BMJ [7].

An early pioneer of this approach was Ramon Llull, a medieval Franciscan monk who postulated that if you had a series of basic "truths," these could be combined to make greater sets of truths that were, de facto, universally acceptable—a form of combinatorics. As a footnote, it's important to state that problems can be cleanly decomposed or factored in this way only if the subproblems are independent of each other.

### Case study

I've finished my undergraduate degree and am looking at where to study my PhD.

Often this decision is expressed as: should I study A at X or B at Y. Clearly, it's very complex to compare these 2 options. In order to not to miss possible solutions, we must avoid considering any aspect in isolation. Thus, although a valid approach is to consider the merits of A over B (if course content is our primary decider), this leads to a de facto answer of institution X or Y. Equally considering X over Y first leads to either A or B. Thus, we have to include all the characteristics of both the course and the institution in the decision matrix.

## Rule 2: Mitigate bias with the right set of inputs

Confirmation bias is a tendency to search for or interpret information in a way that confirms existing preconceptions. This means you put more weight on things you implicitly agree with and less on those that appear to go against your initial main thrust of a "good" decision. It is easy to unbalance the whole process if you start off with a predetermined idea and ignore key elements. Guarding against this is very complex and the subject of much academic study.

In practice, assembling and measuring the objective elements of decisions is usually straightforward—you can look up data—but you have to be measuring the right sets of elements. Most areas have, at the very least, quasi-objective data easily available, e.g., professional or reputable reviewers, specialist journalists, etc., and it's worth looking to see that the elements that third parties might commonly use to appraise topics are also in your own review.

### Case study

I always thought that a top university is the place to study—is that true?

Many worldwide university rankings would support this opinion, or even have given rise to it. However, academic cultures vary from nation to nation and so do the teaching approaches. To take just one example, French scientific institutions tend to produce graduates with a very strong grounding in mathematics and these graduates often go on to excel in research departments worldwide. Therefore, it is a good idea to dig deeper than a university’s general ranking when choosing which institution to attend. This means finding out what the reputation of a particular course or research group of interest is. Especially in research, the reputation of a particular lab may well differ starkly to the department’s or even the university’s reputation as a whole.

## Rule 3: Greater transparency helps in making the right decisions

Most decisions are influenced by others even if they aren’t made with direct external input. By breaking down your decision into components (a number of which may be motivations) and sharing that breakdown, it's simpler to demonstrate reasoning, and it can then be more straightforward to persuade others your decision is the right one.

### Case study

This is really a restatement of the idea most of us became familiar with early on: it's not enough to get the right answer, you must also show your methods.

By exposing the workings, you avoid a "black box," and this can be incredibly useful when asking others to check your work for errors or simply asking them to follow your logic. For example, if you are administering staff and want to introduce a new workplace rule, explain why during consultations—that way, people are more amenable to accepting the underlying logic, and it can make it easier to negotiate problems as you can focus on resolving any minor issues without derailing the whole idea.

## Rule 4: Feedback loops should be utilized to improve future decision-making and inform others

By breaking down and codifying your reasoning, you can come back to it after the decision has played out. Did I make the right choices? And if not, what did I assume that was wrong in the original reasoning. Hindsight is wonderful if applied properly.

We make decisions with a surprising variation in frequency—trivial ones are made daily (what shall I have for lunch?), others yearly (where shall we vacation?), every several years (let's replace the car), or a few times in life (which property shall we buy; shall I take this job?). Many of these are repeated, so it's good not to repeat obvious errors. A few decisions are unique of course (what shall I study at university?).

But even trivial decisions are worth considering more closely. If you want to eat healthily, it's worth thinking objectively about your lunch habits, and if you were only partly satisfied with your last car, why was that? You can't do much about your choice of subject to study at university, but you can tell others about the results of your choice, and that helps inform them.

### Case study

For my PhD, I chose an emerging rather than an established field. How well did that work for me? If it worked well, I might consider the same approach for a postdoctoral position. If the risk paid off (or not), the experience should be shared.

## Rule 5: Get multiple opinions

In Rule 2, we considered bias. Multiple diverse expert opinions tend to mitigate bias. Do not be reticent in getting those opinions—your future may depend on it. Once obtained, consider all opinions carefully. A key word in this short rule is "expert"—on the internet everyone has an opinion, and of course, that is valid for them. But it might well not be very objective. So, take some time to consider the experience and qualifications of the source of the opinion.

### Case study

You are considering a field of study for your PhD, let’s say, cell signaling. If you ask a cell signaling researcher, they will inevitably tell you it is an admirable field of study—we tend to create students in our own image. So, ask other scientists from different fields for their view and weigh all advice carefully.

## Rule 6: Some decisions aren't yours to make alone

In the corporate and academic worlds, decisions are often made by groups, and we often bring to mind the old truism that "a camel is a horse designed by a committee." People involved in these decisions come at them with a varying degree of bias and subjectivity (for example, is this good for my department rather than the university as a whole). Again, by breaking decisions into discrete parts, in this case, per individual, such bias is more obvious, and it is more amenable to be settled objectively, and some level of consensus may emerge.

### Case study

Along with colleagues, you may have shortlisted 3 candidates for a tenure-track position. But what if they don't agree with your choice?

You will have to argue both the facts and perhaps the emotion. You can then separate the fact part of the discussion from the emotional side and treat these appropriately, perhaps by going through the decision process step-by-step.

## Rule 7: Beware of cognitive dissonance

Cognitive dissonance is the psychological stress experienced by a person who holds 2 or more contradictory beliefs, ideas, or values [8]. People experiencing such conflicts generally try to reduce the psychological discomfort by attempting to achieve emotional equilibrium in a variety of ways, for example, downplaying, outweighing, repressing, or incorporating information. When they put these beliefs under stress (for example, in decision-making), they can experience problems, especially if they receive new information that clashes with an existing belief or notion.

These issues all mitigate against good decision-making. But it's worthwhile noting that real dilemmas can occur and they are worth flagging if they do. It's an interesting side note to recognize that a lemma is a small truth, a stepping stone on the way to a larger truth (a theorem). So, a dilemma is just 2 opposite truths, and you can have a trilemma as well. Again, by breaking a decision down into component parts, we help to recognize the presence of such dilemmas and find ways to resolve them.

### Case study

You have a parent whose graduate studies were in A, and they would like you to follow in their footsteps. But field B really stands out, and this is why you prefer it.

If you simply rely on facts, you will never get to the emotional feelings being expressed by the parent, so you have to connect the 2. One good way to do this is to ask open-ended questions (ones that cannot be answered with a yes or no) and to argue the facts or to present a fact and ask the parent how this might make them feel. Emotions are real and have to be dealt with. Objectivity only goes so far.

## Rule 8: Not all systems are amenable to objective decisions

When a decision is tied to strong emotional elements, you have to decide if objectivity is the right way to satisfy yourself in the longer term. You will have to live with your choice and perhaps deal with the psychological repercussions. It's one thing in the cold light of day to decide A, B, and C are the things you most like about, say, a city to go and live or work in, but several years on, you may well wonder why you never felt really at home there.

### Case study

A typical example of this are human partnerships in which both people are pursuing equally important but different careers. For one party, the ideal place to work might be A, whereas for the other party it is B (A and B being in different cities or countries). There may be no objective answer to this.

## Rule 9: What if? Working backwards from a range of solutions

"What if" scenarios are in which a range of possibilities are modeled to look at possible outcomes. After a "base model" is produced, inputs are then varied, and outputs scrutinized. Weightings are then varied, and how this changes the decision can be reviewed. This gives you a very good idea about what is driving a decision in a certain way and may allow room to reflect further on your original choices of key important elements. However, it should be mentioned that blind "goal seeking" by manipulating inputs is generally a terrible way to make a choice and also that this is not a simple binary decision tree type of outcome (if this: then that).

### Case study

When thinking about a university for graduate studies, there are a set of options (outputs). Features include various ranking criteria, the prestige of the lab you are joining, the labs current productivity, etc. Weighting those factors by what is important to you will point you to a solution.

## Rule 10: Apply the weighted decision matrix

And so, to Rule 10—a way to easily increase objectivity in decision-making. For those interested in history, Pascal's Wager is often considered the first example of decision theory [9]. Nowadays, however, decision matrices will be a tool familiar to anyone who has come across Six Sigma (6σ), a set of techniques and tools for process improvement in industry [10], and the Pugh Matrix [11], a more generalized description of the process.

At their most basic, these matrices require you to break a decision down to a set of discrete elements or criteria and then compare these to each other, which gives a level of internal consistency. Weighting can then improve the results further.

There is a large body of literature on decision theory and choice under uncertainty, and although it is unrealistic to try to cover it in this short article, it should be mentioned that it has been a subject of study for centuries and has led to several Nobel prizes in areas such as expected utility theory and prospect theory.

### Use case

Every lab needs a good coffee machine, so let’s use this simple example—you need a new "pod"-based coffee machine. We can start by comparing models from companies like Lavazza, Nespresso, Keurig, etc. We then break down the elements of these (for example, price of machine, price of pods, availability, quality of coffee, aesthetics of machine, etc.). Many of these elements are amenable to fact-based comparisons (price, for example), whereas others are amenable to what we might call metafacts (quality of coffee, where you can read reviews by coffee experts or simply try them yourselves), and others are essentially subjective (e.g., aesthetics). We can then assign values (e.g., from 0–10).

**Fig 1:**
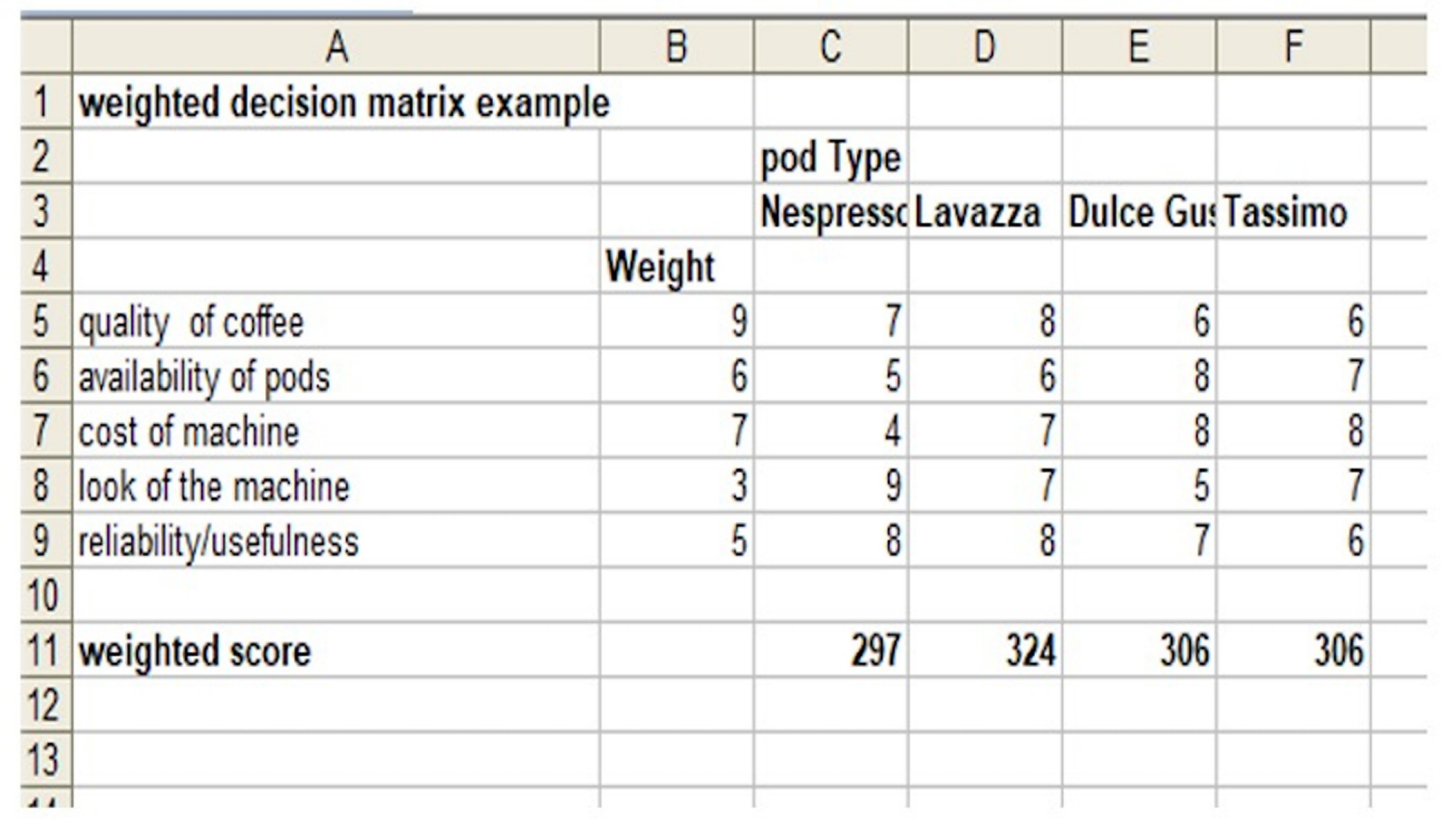
A simple weighted matrix.

When this matrix is complete, we then add another column that applies a weighting: so, if you rate aesthetics more than coffee quality or don't care much about price, for example, you can reflect this. A simple calculation then provides a "winner." It's also very easy to do a "what if" on this by varying weightings. An example of this would be if I cared a bit more about the quality and little less about the price, or alternatively, it might be an ugly machine, but the coffee is great (Fig 1).

The range of uses for this type of analysis are wide, from deciding where to pursue your PhD or postdoc to deciding upon which house or car to buy.

The more the elements lend themselves to data, or metadata, the more useful they are, but it's not an exact tool. Subjectivity is being brought to many aspects, not least the weightings or even the selection of elements. However, because it's quick to do, it can provide useful pause for reflection when it comes to bigger decisions.

## Conclusion

These 10 rules consider, in a simplistic way, features and tools to help make decisions, career related or otherwise. We aren't arguing that fully objective decision-making is possible or even desirable. What we do argue is that a little time spent breaking down the elements of a decision leads to useful insights, and we outline a simple tool that is helpful in this process.

Abraham Lincoln was known to exercise objectivity by writing letters he never sent: the process of simply preparing an argument and committing it to paper is cathartic in itself, and it can also give unexpected insight into the issue at hand [12]. We simply extend that idea here.

Best of luck finding the best coffee machine for your lab.
